# Spatiotemporal Heterogeneity of Tumor Glucose Metabolism Reprogramming: From Single-Cell Mechanisms to Precision Interventions

**DOI:** 10.3390/ijms26146901

**Published:** 2025-07-18

**Authors:** Xiaoxue Chai, Qian Tao, Lili Li

**Affiliations:** Cancer Epigenetics Laboratory, Department of Clinical Oncology, State Key Laboratory of Translational Oncology, Sir YK Pao Center for Cancer, The Chinese University of Hong Kong, Shatin, Hong Kong; xiaoxuechai@cuhk.edu.hk

**Keywords:** spatiotemporal heterogeneity, tumor, glucose metabolism reprogramming

## Abstract

Glucose metabolism reprogramming as a defining hallmark of cancer has become a pivotal frontier in oncology research. Recent technological advances in single-cell sequencing, spatial omics, and metabolic imaging have transformed the field from static bulk analyses to dynamic investigations of spatiotemporal heterogeneity at a single-cell resolution. This review systematically summarizes the current knowledge on tumor glucose metabolism dynamics, discussing spatial heterogeneity and temporal evolution patterns, metabolic subpopulation interactions revealed by single-cell metabolomics, the glucose metabolism–epigenetics–immunology regulatory axis, and therapeutic strategies targeting metabolic vulnerabilities. Recent technological advances in single-cell sequencing and spatial omics have transformed our understanding of tumor glucose metabolism by providing high-resolution insights into metabolic heterogeneity and regulatory mechanisms, contrasting with classical bulk analyses. Spatiotemporal heterogeneity critically influences therapeutic outcomes by enabling tumor cells to adapt metabolically under selective pressures (e.g., hypoxia, nutrient deprivation), fostering treatment resistance and relapse. Deciphering these dynamics is essential for developing spatiotemporally targeted strategies that address intratumoral diversity and microenvironmental fluctuations. By integrating cutting-edge advances, this review deepens our understanding of tumor metabolic complexity and provides a conceptual framework for developing spatiotemporally precise interventions.

## 1. Introduction: Paradigm Shift in Tumor Glucose Metabolism Research

From Warburg’s aerobic glycolysis discovery to current single-cell metabolic mapping, cancer metabolism research now reveals spatiotemporal heterogeneity and therapeutic vulnerabilities. The field has progressed from classical enzymology and bulk metabolic analyses to single-cell transcriptomics, enabling a systems-level metabolic–immune–epigenetic network. This progress has facilitated the clinical development of targeted metabolic drugs, with numerous agents now in ongoing oncology trials.

This review summarizes the current advances in tumor glucose metabolism research, presenting three new insights: a single-cell metabolic topology mapping glycolytic network complexity; the glucose metabolism–epigenetics–immune axis as a multilayer regulatory circuit; and temporal targeting for metabolic oscillations. By integrating multi-omics data with clinical insights, we connect mechanistic understanding to therapeutic development, advancing next-generation metabolic interventions.

## 2. Multidimensional Characteristics of Spatiotemporal Heterogeneity in Tumor Glucose Metabolic Reprogramming

**Figure 1:**
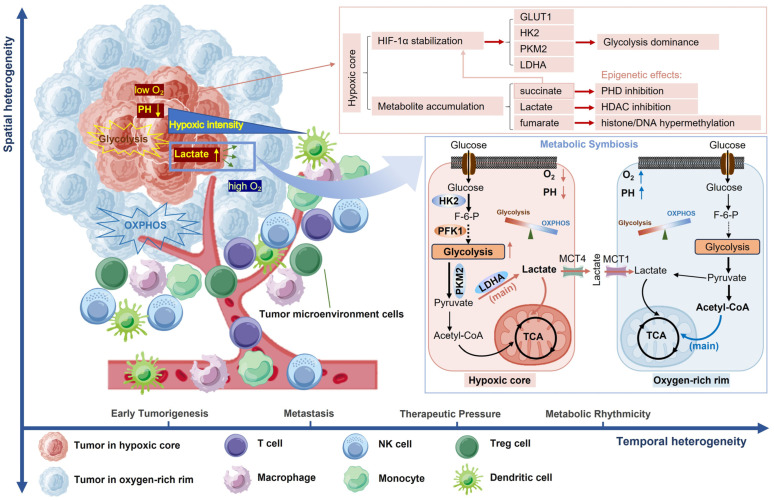
Spatiotemporal heterogeneity of tumor glucose metabolism. This schematic illustrates the dynamic interplay of spatial and temporal heterogeneity in tumor glucose metabolism, driven by microenvironmental gradients (e.g., oxygen, pH, metabolite). Spatially, tumors exhibit metabolic zonation: a hypoxic core characterized by severe oxygen deprivation, decreased pH, HIF-1α stabilization, and lactate accumulation, predominantly reliant on aerobic glycolysis (the Warburg effect); contrasted with an oxygen-rich rim characterized by adequate oxygen supply and relatively higher pH compared to the core, dependent on OXPHOS for efficient energy production. The metabolic symbiosis between these regions is mediated by lactate shuttling via monocarboxylate transporters (MCT1/MCT4), promoting tumor survival and invasion. Additionally, tumor-immune metabolic crosstalk further modulates the microenvironment. Temporally, heterogeneity arises from distinct tumor progression stages (e.g., glycolysis-driven early growth vs. mitochondrial reactivation in metastasis), therapy-induced metabolic plasticity, and intrinsic metabolic rhythms. Tumor spatiotemporal adaptability facilitates therapeutic evasion by dynamically reshaping the metabolic landscape, contributing to treatment resistance and relapse. Elucidating these mechanisms is crucial for advancing precision oncology strategies to counteract compensatory adaptations and intratumoral heterogeneity, such as spatially targeted metabolic inhibitors or rational combination therapies. HIF-1α, hypoxia-inducible factor 1 alpha; OXPHOS, oxidative phosphorylation; MCT1/MCT4, monocarboxylate transporter 1/4. Graphical Elements from BioGDP (https://biogdp.com) [1].

Tumor glucose metabolism reprogramming exhibits spatiotemporal heterogeneity characterized by microenvironmental pressures and cellular signaling. Understanding these regulatory mechanisms enables targeted metabolic therapies. This section analyzes glucose metabolic heterogeneity across tumor evolution and its role in treatment resistance and immune modulation. Figure 1 shows the spatiotemporal heterogeneity of tumor glucose metabolism.

Single-cell transcriptomics provides an unprecedented resolution, revealing intricate metabolic zonation within tumors—far beyond the limitations of bulk measurements in classical studies. By dissecting the cell-to-cell variability, this approach highlights how microenvironmental gradients (e.g., hypoxia, nutrient availability) shape distinct metabolic phenotypes, offering new mechanistic insights into tumor metabolic adaptation.

### 2.1. Spatial Heterogeneity

#### 2.1.1. Intratumoral Metabolic Zonation

Tumors establish spatially structured metabolic networks shaped by oxygen and nutrient gradients. Oxygen-rich rims utilize oxidative phosphorylation (OXPHOS), along with accumulated tricarboxylic acid (TCA) cycle intermediates (citrate, malate, succinate) and glutaminase 1 (GLS1)-dependent glutamine catabolism, to meet both the biosynthetic needs and invasive capacity [2,3]. Conversely, hypoxic cores exhibit hypoxia-inducible factor 1 alpha (HIF-1α)-dependent metabolic reprogramming by glycolytic dominance (elevated lactate/pyruvate) [2] and the suppression of oxidative metabolism via lactate dehydrogenase A (LDHA)-driven pyruvate diversion [4,5]. In high-grade osteosarcomas, hypoxic cores selectively upregulate hypoxia-inducible factor 2 alpha (HIF-2α) (not HIF-1α) to activate proline dehydrogenase (PRODH)-dependent proline metabolism, maintaining the redox balance under nutrient stress [6]. Pancreatic neuroendocrine tumors (PanNETs) lack metabolic zonation due to dense vascularization, showing homogeneous glycolytic phenotypes throughout via aberrant mammalian target of rapamycin (mTOR)-vascular endothelial growth factor (VEGF) crosstalk [7]. Spatial transcriptomics in glioblastoma revealed a progressive decline in hypoxia-responsive genes (e.g., *vascular endothelial growth factor A* (*VEGFA*)) and glycolytic genes (e.g., *glucose transporter 1* (*GLUT1*)) with increasing distance from necrotic regions [8], demonstrating how metabolic adaptation correlates with microenvironmental topography. This compartmentalization creates therapeutic challenges, as regions may exhibit differential sensitivity to metabolic interventions (see Table 1 for cross-tumor comparisons).

#### 2.1.2. Metabolic Symbiosis Networks

Metabolic symbiosis refers to cooperative metabolic interactions between distinct tumor cell populations or between tumor cells and stromal components, where metabolic byproducts or substrates are exchanged to fulfill complementary bioenergetic or biosynthetic demands. Tumors exhibit oxygen-dependent metabolic zonation, creating symbiotic networks between compartments. Hypoxic cells export glycolysis-derived lactate via the low-affinity monocarboxylate transporter 4 (MCT4), while oxygen-rich cells uptake it via the high-affinity monocarboxylate transporter 1 (MCT1) for mitochondrial OXPHOS (the reverse Warburg effect) [9,10]. Beyond its bioenergetic role, this “reverse Warburg effect” creates a pro-tumorigenic niche through lactate-driven VEGF-mediated angiogenesis and acidification. Single-cell studies in esophageal squamous cell carcinoma (ESCC) have uncovered metabolite exchange with cytotoxic T cells and pro-tumor M2 macrophages, but the metabolic exclusion of B cells [11], highlighting immunometabolic crosstalk in tumor progression.

Tumor ecosystems additionally employ stromal metabolic support, where cancer-associated fibroblasts (CAFs) and tumor-associated macrophages provide alternative metabolic substrates (e.g., glutamine, ketone bodies) but competitively consume glucose and arginine to impair immune function [11]. Cancer stem cells (CSCs) display OXPHOS-dominant, low-glycolysis phenotypes to maintain their stem-like properties, further contributing to tumor metabolic heterogeneity. Single-cell metabolomics has identified therapy-resistant fatty acid oxidation (FAO)-dependent clones coexisting with glycolytic proliferative populations in breast and esophageal cancers [11]. These findings highlight metabolic symbiosis as a multifaceted driver of tumor progression and therapeutic resistance.

**Table 1:** Comparison of spatial metabolic characteristics of different types of tumors.

Tumor Type	Metabolic Characteristics of the Core Region	Metabolic Characteristics of the Marginal Zone	Key Molecules of Metabolic Interactions	Clinical Significance
Glioblastoma [12]	Enhanced glycolysis and hypoxia-induced HIF-1α	OXPHOS is active and aggressive.	LDHA, MCT4	Hypoxic regions are resistant to radiotherapy.
Glioblastoma [13]	Significant glycolytic phenotype, high expression of hypoxia-related genes, significant chromosomal copy number variations (CNAs).	The metabolic state is more similar to that of normal tissues and more dependent on oxidative phosphorylation. Infiltration of T cells and myeloid cells was observed, but the degree of immunosuppression was low.	HIF-1α, VEGFA	Multimodal treatment: core zone (glycolysis inhibitor + radiotherapy) combined with marginal zone (OXPHOS inhibitor + immunotherapy).
OSCC [14]	Energy is obtained mainly through aerobic glycolysis, which produces large amounts of lactic acid.	Immune cells and stromal cells in the TME take up lactate to produce energy.	HIF-1α, CXCL12, TGF-β	Combined targeting of lactic acid metabolism (such as MCT inhibitors) and immune checkpoint inhibitors may enhance the therapeutic effect.
OSCC [15]	Significant glycolytic activity was enhanced, with localized enrichment of glycolytic metabolites.	The retention of higher glucose levels may serve as a “reservoir” of glucose to support tumor metabolic requirements.	Hexokinase, pyruvate kinase	Targeting key glycolytic enzymes (such as hexokinase and lactate dehydrogenase) or the inhibition of glycolytic pathways (such as the use of 2-deoxyglucose) may inhibit the tumor energy supply, especially for OSCC subtypes that are resistant to conventional chemoradiotherapy.
Cervical squamous cell carcinoma [16]	OXPHOS activity was low and hypoxia-related metabolic pathways dominated.	The OXPHOS pathway was significantly activated.	PRKCE, ITGA2, PKM	A combination strategy is appropriate.
Gastric cancer [17]	Glycolytic activity was highest and lactate accumulation was accompanied by the upregulation of tricarboxylic acid cycle intermediates, such as succinate.	Some regions may retain OXPHOS capacity, but are affected by tumor–stroma interactions and have complex metabolic phenotypes (e.g., immune cell-infiltrating regions may inhibit glycolysis).	PKM2, PFKL, ENO1	Early screening and staging based on glycolysis imaging; combined strategies targeting glycolysis (e.g., PFKFB3 inhibitors) with immunometabolic regulation (e.g., GLS inhibitors).
Breast cancer [18]	The glucose content is high and more prone to glycolytic metabolism.	With a preference for mitochondrial metabolism.	PI3K, GLUT1	Combined inhibition of PI3K and the bromodomain can overcome drug resistance and reduce metabolic heterogeneity.
Osteosarcoma [6,19]	Nucleotide/amino acid pathway amplification (alanine, aspartate, proline); glycolytic dominance; mitochondrial dysfunction.	FAO-enhanced invasion; ACLY-mediated acetyl-CoA production.	HIF-2α, PRODH, CPT1A	PRODH inhibitors sensitize to hypoxia-targeted therapy.
Leiomyosarcoma [20,21]	Highest glycolytic activity; lactate accumulation.	Retained OXPHOS capacity; modulated by tumor–stroma interactions.	RAS/PI3K pathway, FBP2	High glycolysis correlates with poor prognosis.
PanNETs [7]	Homogeneous glycolysis (mTOR-VEGF axis dominance); under hypoxic conditions, invasive PanNET achieved metabolic adaptation by upregulating glycolysis and downregulating oxidative phosphorylation.	Lactate shuttling to stromal fibroblasts.	mTOR, VEGF, MCT4, HDAC1/2	mTOR inhibitors reduce glycolytic flux but increase metastasis risk.
PDAC [22]	Enhanced glycolysis, hypoxia-induced HIF-1α stabilization, lactate accumulation, upregulation of glucose transporters (e.g., GLUT1), and key glycolytic enzymes (e.g., HK2, PFK1, LDHA).	OXPHOS activity is present but often compromised due to tumor–stroma interactions, lactate uptake via MCT1.	HIF-1α, GLUT1, HK2, PFK1, LDHA, MCT1, MCT4, CD147	Hypoxic regions are resistant to radiotherapy; metabolic heterogeneity contributes to treatment resistance and relapse; targeting metabolic vulnerabilities (e.g., glycolysis inhibitors, MCT inhibitors) may enhance therapeutic efficacy.

Note: HIF-1α, hypoxia-inducible factor 1 alpha; OXPHOS, oxidative phosphorylation; LDHA, lactate dehydrogenase A; MCT4, monocarboxylate transporter 4; VEGFA, vascular endothelial growth factor A; OSCC, oral squamous cell carcinoma; TME, tumor microenvironment; CXCL12, C-X-C motif chemokine ligand 12; TGF-β, transforming growth factor beta; PRKCE, protein kinase C epsilon; ITGA2, integrin subunit alpha 2; PKM, pyruvate kinase M; PFKL, phosphofructokinase, liver type; ENO1, enolase 1; PFKFB3, 6-phosphofructo-2-kinase/fructose-2,6-biphosphatase 3; PI3K, phosphoinositide 3-kinase; GLUT1, glucose transporter 1; FAO, fatty acid oxidation; ACLY, ATP citrate lyase; HIF-2α, hypoxia-inducible factor 2 alpha; PRODH, proline dehydrogenase; CPT1A, carnitine palmitoyltransferase 1A; FBP2, fructose-1,6-bisphosphatase 2; PanNET, pancreatic neuroendocrine tumor; mTOR, mechanistic target of rapamycin (or mammalian target of rapamycin); VEGF, vascular endothelial growth factor; HDAC1/2, histone deacetylase 1/2; PDAC, pancreatic ductal adenocarcinoma; HK2, hexokinase 2; PFK1, phosphofructokinase 1.

#### 2.1.3. Organ-Specific Metabolic Adaptation of Metastatic Lesions

Metastatic tumors exhibit remarkable metabolic plasticity by adapting to organ-specific microenvironmental conditions through distinct reprogramming strategies. Liver metastases exploit high lactate concentrations by upregulating MCT1 and lactate dehydrogenase B (LDHB) to convert lactate into pyruvate for the TCA cycle anaplerosis [23]. Oxygen-rich lung metastases preferentially utilize OXPHOS and glutaminolysis to overcome glucose limitation. Cerebral metastases circumvent blood–brain barrier restrictions by metabolizing astrocyte-derived ketone bodies [24]. Interestingly, organ-specific metabolic adaptations show striking contrasts between pancreatic and colorectal metastases. Pancreatic metastases exhibit mitochondrial dysfunction through mitochondrial DNA (mtDNA) mutations (e.g., Complex I/III defects) that reduce OXPHOS capacity, forcing glycolytic dependence, although a subset of CSCs maintain elevated OXPHOS activity via peroxisome proliferator-activated receptor gamma coactivator 1-alpha (PGC-1α) to preserve stemness [25,26]. Conversely, colorectal metastases display metabolic compartmentalization: liver lesions develop glycolytic edges (lactate dehydrogenase A (LDHA)/hexokinase 2 (HK2)) for invasion but OXPHOS/glutamine synthetase (GLUL)-dependent cores for hypoxic survival via HIF-1α/pyruvate dehydrogenase kinase 4 (PDK4) [27], while lung metastases upregulate carnitine palmitoyltransferase 1A (CPT1A)-driven FAO to exploit alveolar niches [28]. This comparison underscores how the primary tumor origin dictates distinct metastatic metabolic programs.

Single-cell metabolomics reveals that breast cancer bone metastases activate osteolytic destruction via parathyroid hormone-related protein (PTHrP)-induced transforming growth factor beta (TGF-β)/calcium signaling, concurrently increasing glycolytic demands [8]. Such adaptations are achieved through two mechanisms: microenvironmental selection and exosome-mediated preconditioning, facilitated by metabolic enzymes (e.g., HK2, LDHA) that remodel distant niches prior to colonization [29]. Thus, metastases develop metabolic diversity through spatial patterns like intratumoral zonation and organ adaptation, and temporal changes including therapy resistance and progression.

### 2.2. Temporal Heterogeneity: Metabolic Dynamic Evolution and Therapeutic Adaptation

**Figure 2:**
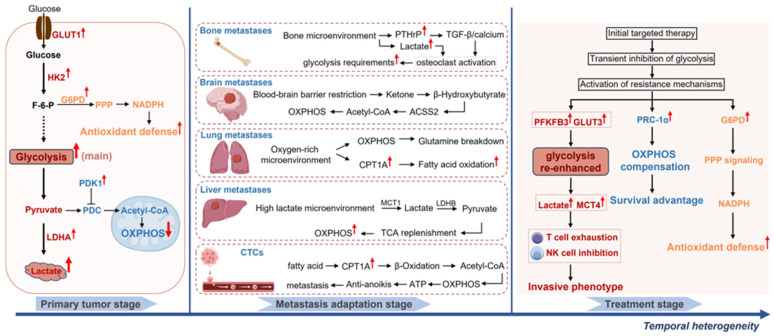
Metabolic reprogramming across tumor progression and therapeutic intervention. This schematic illustrates dynamic metabolic adaptations during three critical phases: (1) Primary tumor expansion, characterized by enhanced glycolysis (GLUT1/HK2 upregulation driving lactate accumulation), PPP activation (G6PD upregulation leading to increased G6PD-mediated NADPH production for antioxidant defense), and enhanced suppression of OXPHOS via upregulation of PDK1 and its inhibition of PDC; (2) Metastatic adaptation, featuring niche-specific metabolic shifts: bone metastasis (PTHrP-TGF-β/calcium signaling driving osteoclast-mediated glycolysis and lactate production), brain metastasis (ACSS2-facilitated efficient ketone utilization via β-hydroxybutyrate-to-acetyl-CoA conversion to promote OXPHOS), lung metastasis (OXPHOS enhancement and CPT1A-dependent fatty acid oxidation in an oxygen-rich microenvironment), liver metastasis (MCT1/LDHB-mediated lactate scavenging to fuel OXPHOS via TCA cycle anaplerosis), and CTCs (CPT1A-driven fatty acid β-oxidation supporting anoikis resistance and metastasis); (3) Therapeutic resistance, where initial glycolysis suppression gives way to resistance mechanisms, including glycolytic rebound causing T-cell exhaustion and NK cell suppression, PRC-1α-mediated OXPHOS compensation promoting tumor cell survival, and G6PD/PPP-driven antioxidant defense via upregulated G6PD activation. GLUT1, glucose transporter 1; HK2, hexokinase 2; PDK1, pyruvate dehydrogenase kinase 1; PDC, pyruvate dehydrogenase complex; OXPHOS, oxidative phosphorylation; PPP, phosphorylative pentose; G6PD, glucose-6-phosphate dehydrogenase; NADPH, nicotinamide adenine dinucleotide phosphate; PTHrP, parathyroid hormone-related protein; ACSS2, acyl-CoA synthetase short-chain family member 2; CTC, circulating tumor cell. Graphical Elements from BioGDP (https://biogdp.com) [1].

Extending our analysis of spatial dynamics, we next investigate the temporal evolution of tumor glucose metabolism across disease progression and therapeutic contexts, focusing on three key processes: metabolic phase transitions, therapy-induced adaptive remodeling, and circadian regulation mechanisms. Figure 2 illustrates the metabolic flux alterations during tumor progression and under therapy.

#### 2.2.1. Metabolic Phase Transitions During Tumor Progression

Tumor metabolism undergoes dynamic temporal evolution during progression. Early tumors adopt aerobic glycolysis (the Warburg effect) to gain selective advantages: rapid adenosine triphosphate (ATP) production for energy demands [30], reduced oxidative stress during hypoxia via nicotinamide adenine dinucleotide (NAD+) recycling [29,31], and microenvironment remodeling that promotes angiogenesis [32]. This metabolic shift promotes tumor growth and suppresses mitochondrial function, facilitating cancer progression. Fatty acids exhibit stage-specific roles, supporting invasion, circulating cell survival, and metastasis. Hypoxia induces glutamine dependence via c-Myc/HIF-2α-mediated upregulation of GLS1 and solute carrier family 1 member 5 (SLC1A5), thereby facilitating mitochondrial oxidation [3,33]. Metastases reactivate mitochondrial metabolism through peroxisome proliferator-activated receptor gamma coactivator 1-alpha (PGC1α)-driven TCA cycle enhancement and glutamine reprogramming [34]. This metabolic rewiring not only meets progression-specific energy and redox demands but also creates therapeutic vulnerabilities at each progression stage, revealing the remarkable adaptability of cancer cells during dissemination.

#### 2.2.2. Metabolic Adaptive Remodeling Under Therapeutic Pressure

Metabolic adaptive remodeling under therapeutic pressure is a major clinical challenge, as targeted therapies can rapidly reprogram tumor glucose metabolism, driving compensatory resistance mechanisms (Table 2). Cancer cells evade therapy primarily by enhancing glycolysis and rewiring mitochondrial function. In gefitinib-resistant *epidermal growth factor receptor* (*EGFR*)-mutant non-small cell lung cancer (NSCLC), lactate is overproduced by the upregulation of GLUT1, HK2, and LDHA [35]. Vemurafenib-resistant *v-Raf murine sarcoma viral oncogene homolog B1* (*BRAF*)-mutant melanomas activate mitogen-activated protein kinase (MEK)/extracellular signal-regulated kinase (ERK) signaling to increase GLUT1/3 and HK2 expression, enhancing glucose uptake and glycolytic flux [36]. Trastuzumab-resistant human epidermal growth factor receptor 2 (HER2+) breast cancers drive glycolysis through 6-phosphofructo-2-kinase/fructose-2,6-biphosphatase 3 (PFKFB3)-mediated fructose-2,6-bisphosphate synthesis [37]. Mitochondrial reprogramming represents another resistance strategy. Osimertinib-resistant NSCLC cells enhance OXPHOS activity [38], and BRAF inhibitor-resistant melanomas activate PGC-1α-mediated mitochondrial biogenesis, further increasing the cristae density and elevating oxidative capacity [39].

Metabolic adaptations also drive immunotherapy resistance. Under programmed cell death protein 1 (PD-1) inhibition, tumors select for clones with enhanced OXPHOS that frequently co-express T cell immunoglobulin and mucin domain 3 (TIM-3), linking metabolic reprogramming directly to immune evasion mechanisms [40]. These findings demonstrate that metabolic plasticity serves as a fundamental mechanism of therapeutic resistance across diverse anticancer treatment modalities.

#### 2.2.3. Circadian Regulation of Tumor Metabolism

Circadian metabolic rhythmicity offers new insights into tumor heterogeneity, with the core clock components (Clock Circadian Regulator (CLOCK), brain and muscle ARNT-like 1 (BMAL1/ARNTL), Period Circadian Regulator (PER)) acting as master regulators of oncogenic metabolism. Dysregulation of core clock genes not only correlates with disease progression and poor prognosis across multiple cancer types [41] but also directly initiates tumor formation by rewriting metabolic processes [41,42]. For example, the *Zinc Finger Protein 704* (*ZNF704*)-*SIN3 Transcription Regulator Family Member A* (*SIN3A*) transcriptional repressor complex destabilizes *period circadian regulator 2* (*PER2*) transcription, destabilizing circadian rhythms and driving breast cancer progression [43]. At the metabolic interface, core clock genes directly regulate metabolic enzymes: BMAL1 or CLOCK knockdown disrupts both mitochondrial respiration and glycolysis [44].

Circadian systems in cancer dynamically interact with microenvironmental stressors. Under hypoxic conditions, BMAL1 stabilizes HIF-1α to facilitate metabolic adaptation, while fluctuating oxygen levels synchronize circadian rhythms via HIF-1α-mediated regulation of clock genes [45]. This reciprocal relationship between circadian and hypoxic signaling establishes a self-reinforcing loop that promotes genomic instability and metabolic plasticity. Thus, circadian disruption emerges as a critical contributor to spatiotemporal tumor heterogeneity, highlighting the potential for time-based metabolic therapies. With this temporal framework established, we next will integrate the spatial and temporal dimensions to elucidate the molecular regulatory networks governing metabolic heterogeneity.

## 3. Regulatory Networks of Spatiotemporal Heterogeneity in Tumor Glucose Metabolism Reprogramming

### 3.1. Spatial Heterogeneity: Topographic Metabolic Landscapes and Microenvironmental Crosstalk

#### 3.1.1. Oxygen Gradient-Driven Metabolic Zonation Mechanism and Epigenetic Interplay

Tumors develop spatially heterogeneous oxygen gradients that establish distinct metabolic zones, with HIF-1α orchestrating the cellular response to hypoxia. Under hypoxia, stabilized HIF-1α dimerizes with HIF-1β to transactivate genes governing glycolysis (*GLUT1/3*, *HK2*, *LDHA*), angiogenesis (*VEGF*), and pH regulation (*Carbonic Anhydrase IX* (*CAIX*), *MCT4*), simultaneously suppressing oxidative metabolism through pyruvate dehydrogenase kinase 1 (PDK1)-mediated inhibition of pyruvate dehydrogenase [46,47,48]. This metabolic reprogramming is further reinforced by crosstalk with oncogenic signaling pathways. The PI3K/Akt/mTOR pathway, for instance, promotes both HIF-1α production and stabilization [49,50].

Metabolic zonation promotes the accumulation of oncometabolites, which coordinate signaling and epigenetic remodeling (Table 3). Lactate promotes epithelial-mesenchymal transition (EMT) via histone lactylation and histone deacetylase (HDAC) inhibition [51,52]. Succinate stabilizes HIF-1α via competitive prolyl hydroxylase (PHD) inhibition [53,54,55], establishing a feedforward hypoxic response. Moreover, succinate and fumarate inhibit alpha-ketoglutarate (α-KG)-dependent dioxygenases (ten–eleven translocation methylcytosine dioxygenases (TETs), histone lysine demethylases (KDMs)), inducing DNA and histone hypermethylation [54]. In addition, 2-hydroxyglutarate disrupts fat mass and obesity-associated protein (FTO)-mediated RNA demethylation [56]. This intricate coupling of oxygen sensing, metabolic reconfiguration, and epigenetic rearrangement establishes a self-reinforcing cycle that sustains tumor heterogeneity, accelerates progression, and confers therapeutic resistance.

#### 3.1.2. Metabolic Competition in the Immunometabolic Microenvironment

Cancer cells escape immune surveillance through metabolic reprogramming. Two key mechanisms drive this process: (1) the depletion of essential nutrients from the tumor microenvironment; and (2) the production of metabolites that suppress immune cell function. Through aerobic glycolysis (the Warburg effect), cancer cells aggressively consume glucose, starving adjacent T cells. This nutrient deprivation disrupts mechanistic target of rapamycin complex 1 (mTORC1) signaling, impairing T-cell activation and proliferation, and interferon gamma (IFN-γ) production [59,60,61].

Lactate accumulation contributes to immune evasion through several mechanisms: (1) impairing dendritic cell function by reducing MHC-II expression and IL-12 secretion via G-protein-coupled receptor 81 (GPR81) [62,63]; (2) expanding regulatory T cells (Tregs) through metabolic–epigenetic reprogramming [64,65]; (3) promoting OXPHOS-dependent Tregs with high PD-1 expression via GPR81/PI3K/AKT/mTOR signaling [64,66], particularly in PD-1-resistant tumors [66,67].

Beyond lactate, other key oncometabolites contribute significantly to immune-suppression within the glucose-deprived tumor microenvironment (TME) and intersect with cancer glucose metabolism. Tumors in the glucose-limited environments enhance glutamine uptake via glutaminase (GLS), converting it to glutamate for TCA cycle replenishment [68]. This glutamine depletion directly impairs T-cell activation and cytotoxicity [69]. Ketone bodies like β-hydroxybutyrate (β-HB) serve as alternative energy sources in glucose-limited conditions. Although certain immune cells (e.g., memory T cells) use β-HB for metabolic homeostasis, its accumulation in the TME may promote immune tolerance independently of HDAC inhibition [70,71]. The hypoxic and nutrient-poor TMEs also accelerate ATP breakdown, elevating extracellular adenosine levels. Through adenosine Receptor A2a (A2aR) binding, adenosine suppresses T-cell and NK cell activity, further promoting immune evasion [72]. Thus, glutamine metabolism supplying the TCA cycle and glycolytic ATP generating adenosine create metabolically linked nodes within tumor glucose metabolism. This interconnected network sustains an immunosuppressive TME and facilitates tumor immune evasion.

Immune evasion is also supported by glycolytic enzymes through non-metabolic mechanisms. The C-terminal domain of HK2 activates NF-κB to upregulate PD-L1 in glioblastoma and breast cancer [73,74,75]. Glycolysis–PI3K/AKT/mTOR crosstalk inhibits CD8^+^ T cells, expands Tregs, and promotes M2 macrophage polarization [76,77,78]. Lactate signaling through MCT4-lactate-PI3Kγ/Akt blocks M1 macrophage polarization via interferon regulatory factor 5 (IRF5) suppression [79]. Thus, these pathways establish an immunosuppressive microenvironment through both metabolic competition and direct immune cell regulation, revealing novel therapeutic targets.

#### 3.1.3. Metabolic Plasticity Drives Tumor Metastasis

Metabolic Adaptations During Invasion and Dissemination

Tumor cells exploit metabolic flexibility to adapt metastasis, from initial invasion through distant colonization. As cells detach from primary sites, hypoxia and nutrient deprivation trigger HIF-1α/2α signaling, enhancing glycolysis and reactive oxygen species (ROS) accumulation, further inducing EMT [80]. Pyruvate kinase M2 (PKM2) reinforces this process by altering nuclear metabolite pools to modulate EMT gene expression [81]. Acidification from lactate and CO_2_ activates MMPs to remodel basement membranes, while glutamine-derived ammonia provides another mechanism driving invasion [82].

Survival Mechanisms of Circulating Tumor Cells (CTCs)

To survive in circulation, circulating tumor cells (CTCs) evade anoikis by upregulating pentose phosphate pathway (PPP)-driven nicotinamide adenine dinucleotide phosphate (NADPH) production to counteract ROS [83,84]. Exosomes mediate metabolic crosstalk: PKM2-containing exosomes from lung cancer cells activate stromal glycolysis, promoting bone destruction [85]. Exosomal miR-122 depletes extracellular glucose to fuel metastasis [86]. Furthermore, tumor exosomes modulate immune metabolism by inhibiting T-cell activation via glycolytic enzyme transfer, whereas glycolysis inhibition conversely restores antitumor immune responses [87]. These adaptations enable CTC survival during dissemination and prime metastatic niches.

Organ-Specific Metabolic Strategies for Colonization

Metabolic reprogramming drives organ-specific patterns of metastatic colonization. Brain metastases utilize acetate (via acyl-CoA synthetase short-chain family member 2 (ACSS2)) and branched-chain amino acids [88,89] in glucose-scarce environments [90]. Lung metastases enhance mitochondrial function through PGC-1α/peroxiredoxin 2(PRDX2) [91,92], favoring pyruvate over glutamine [93]. Liver metastases employ glycolysis and phosphocreatine cycling (PDK1/creatine kinase B-type (CKB)) for rapid ATP production [94,95]. Bone metastases actively remodel the niche through lactate and serine secretion, and drive osteoclastogenesis [88,96,97]. These organ-specific strategies highlight the role of metabolic reprogramming during the spread of metastases.

### 3.2. Temporal Dynamic Regulation: From Genetic Mutations to Epigenetic Memory

Following our analysis of spatial–microenvironmental interactions, we explore temporal–genetic mechanisms, analyzing how mutations and epigenetic modifications drive metabolic adaptations throughout tumor evolution and therapy, with a particular focus on clonal selection, treatment-induced remodeling, and molecular-level circadian regulation.

#### 3.2.1. Metabolic Clonal Selection During Tumor Evolution

During early tumor development, oncogenic drivers like KRAS mutations increase the expression of glycolytic enzymes (HK2, PKM2) to induce the Warburg effect [98]. This metabolic shift favors glycolysis over oxidative phosphorylation, enabling rapid ATP production and generation of biosynthetic precursors (lipids, nucleotides), even in oxygen-rich conditions. This metabolic rewiring further promotes clonal expansion by some mechanisms: (1) glycolytic intermediates (e.g., PPP-generated NADPH) decrease oxidative damage and DNA damage [99]; and (2) p53 loss increases PPP flux through TP53-induced glycolysis and apoptosis regulator (TIGAR) downregulation and glucose-6-phosphate dehydrogenase (G6PD) depression, to enhance NADPH/glutathione (GSH) production [100]. The newly acquired metabolic phenotype confers both biosynthetic efficiency for proliferation and antioxidant protection against microenvironmental stress (hypoxia, inflammation), enabling early clonal dominance and malignant transformation.

In late-stage metastasis, EMT induces profound metabolic changes to facilitate cancer cell dissemination. Transcription factors such as Snail and Twist drive a glycolytic shift by suppressing mitochondrial genes (e.g., *Cytochrome C Oxidase Subunit 4I1*) to reduce OXPHOS and ROS but enhancing glycolysis for migration energy needs [101]. This metabolic rewiring promotes invasiveness through LDHA-mediated lactate production (activating matrix metalloproteinases (MMPs) for extracellular matrix (ECM) degradation) and glycolytic intermediate-supported cytoskeletal remodeling [102]. Emerging evidence, however, reveals substantial complexity in the OXPHOS–metastasis relationship, as many cancers maintain functional OXPHOS alongside glycolysis despite EMT-mediated suppression, including pancreatic ductal adenocarcinoma (PDAC), high-OXPHOS melanoma, and endometrial carcinomas [103,104,105]. This metabolic flexibility helps tumors adapt to microenvironmental changes during metastasis. It has been identified that OXPHOS inhibition enhances metastatic potential in specific biological contexts due to the mutational inactivation of tumor suppressors, such as the p53 mutation affecting mitochondrial function [106,107], EMT-related repression of cytochrome c oxidase via Snail [108], and deletion of KiSS-1 metastasis-suppressor (KISS1)-mediated PGC1α regulation [109]. Conversely, OXPHOS remains essential for metastatic colonization in other contexts: melanoma metastases require OXPHOS/PGC1α for lung/brain colonization [110] and leukemia stem cells depend critically on OXPHOS for persistence [111,112]. This functional duality explains the contradictory findings: OXPHOS suppression may facilitate early dissemination via glycolytic activation but impair colonization in OXPHOS-dependent tumors.

To overcome detachment stress, CTCs activate FAO through the peroxisome proliferator-activated receptor gamma (PPARγ)/CPT1A pathway. The roles of FAO in metabolic adaptation include: (1) generate ATP for energy needs during circulation [113,114]; (2) produce acetyl-CoA for histone acetylation of pro-survival genes (e.g., *B-cell lymphoma 2* (*BCL-2*)) [115,116]; and (3) maintain mitochondrial membrane potential to suppress anoikis [28]. This FAO-dependent adaptation, combined with the initial glycolysis-driven EMT program that enables invasion, creates a coordinated metabolic cascade that drives successful metastatic progression.

#### 3.2.2. Treatment-Induced Metabolic and Epigenetic Remodeling Mechanisms

Cancer cells evade therapy resistance by rewriting metabolic and epigenetic programs. These adaptations establish regulatory networks that manage stress and transmit drug-resistant phenotypes to daughter cells via epigenetic memory, ultimately leading to treatment failure and tumor recurrence.

Chemotherapy causes oxidative stress and DNA damage, forcing cancer cells to survive via metabolic adaptation. Drug-resistant cells combat oxidative stress by activating the nuclear factor erythroid 2-related factor 2 (NRF2)/G6PD axis to boost PPP-derived NADPH for ROS detoxification [117,118,119,120,121]. Platinum-based chemotherapy induces metabolic reprogramming by suppressing serine synthesis. Phosphoglycerate dehydrogenase (PHGDH) downregulation maintains the cellular NAD^+^ levels required for PARP-dependent DNA repair processes. In contrast, 5-FU-resistant cells adapt by upregulating Serine Hydroxymethyltransferase 2 (SHMT2) to enhance serine metabolism and maintain nucleotide production [122,123]. These adaptive changes underscore metabolic plasticity as a primary mechanism of resistance.

Ionizing radiation drives radioresistance through an integrated network of metabolic adaptations. HIF-1α upregulates GLUT1, which enhances glucose uptake, and p53 suppression enhances hypoxic survival [124,125]. ATM Serine/Threonine Kinase (ATM)/NRF2 activates PPP via G6PD for NADPH-mediated ROS clearance and DNA repair [126]. Radiation-induced lactate efflux via MCT1 acidifies the microenvironment, impairing CD8^+^ T-cell function and activating HIF-1α/myeloid-derived suppressor cells (MDSC)-mediated immunosuppression [126]. Concurrently, signal transducer and activator of transcription 5 (STAT5) induces glutamine synthetase to fuel nucleotide synthesis [127]. These synergistic adaptations establish diagnostic metabolic signatures in recurrent tumors [128].

Cancer therapies induce epigenetic–metabolic crosstalk through multiple mechanisms. DNA-damaging drugs modulate the expression of metabolic enzymes by activating DNA methyltransferase 3 alpha (DNMT3A). For example, *isocitrate dehydrogenase 1* (*IDH1*) silencing by promoter methylation promotes glutamine metabolism [129]. *LDHA* or *phosphoenolpyruvate carboxykinase 1* (*PCK1*) hypomethylation enhances glycolytic/gluconeogenic flux [130]. Enhancer of zeste homolog 2 (EZH2)-mediated H3K27me3 regulates chromatin accessibility [131], but its inhibition (e.g., GSK126) disrupts mitochondrial function and triggers glycolytic compensation [131]. This epigenetic plasticity facilitates metabolic adaptation to treatment stress.

#### 3.2.3. Molecular Basis of Metabolic Circadian Rhythm

**Figure 3:**
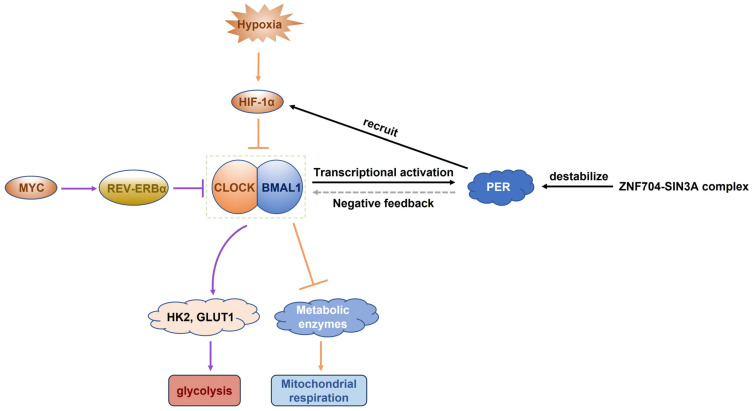
Molecular crosstalk between circadian rhythm disruption and tumor metabolic reprogramming. The core circadian oscillator, the CLOCK-BMAL1 heterodimer, positively regulates *PER* gene transcription. The accumulated PER protein forms an inhibitory feedback loop by suppressing CLOCK-BMAL1 activity, thereby maintaining a 24-h circadian rhythm (gray arrow). PER recruits HIF-1α, and the hypoxic tumor microenvironment stabilizes HIF-1α, enabling cooperative suppression of CLOCK–BMAL1 heterodimer activity. This dual repression concurrently enhances glycolysis and impairs mitochondrial respiration. Conversely, oncogenic metabolic reprogramming involves MYC-driven transcriptional repression of *BMAL1* by REV-ERBα, leading to the upregulation of glycolytic enzymes (HK2 and GLUT1) and enhanced glycolysis (purple pathway). Simultaneously, the ZNF704–SIN3A complex destabilizes PER2, exacerbating circadian disruption and reinforcing metabolic reprogramming. CLOCK, circadian locomotor output cycles kaput; BMAL1, brain and muscle ARNT-like 1; PER, period circadian regulator; HIF-1α, hypoxia-inducible factor 1 alpha; REV-ERBα, nuclear receptor subfamily 1 group D member 1; HK2, hexokinase 2; GLUT1, glucose transporter 1; ZNF704, zinc finger protein 704; SIN3A, SIN3 transcription regulator family member A; PER2, period circadian regulator 2.

Tumor cells disrupt circadian metabolic regulation through multiple mechanisms, including clock gene dysregulation, temporal heterogeneity of metabolic enzyme expression, and time-dependent crosstalk between microenvironmental signals, ultimately contributing to tumor proliferation, metastasis, and resistance. Figure 3 shows the molecular crosstalk between circadian rhythm disruption and tumor metabolic reprogramming.

The deregulation of core clock genes drives tumor progression. BMAL1 deficiency activates cancer-associated fibroblasts, promoting invasion and immune escape [132,133]. PER2 recruits HIF-1α to suppress mitochondrial function, forcing glycolytic metabolism [134]. MYC-induced nuclear receptor subfamily 1 group D member 1 (REV-ERBα) represses BMAL1, upregulating HK2 and GLUT1 [42,135,136]. BMAL1/PER2 knockout accelerates lung tumor growth with glycolytic upregulation [137]. Post translationally, CLOCK phosphorylation at Ser106 promotes nucleotide biosynthesis and tumor proliferation [138]. *Period circadian regulator 1* (*PER1*) mutations enhance glycolytic flux and tumor progression via the receptor for activated C kinase 1 (RACK1)/PI3K/AKT pathway [42,139]. These changes rewire the cancer metabolism, creating a permissive environment for progression.

Tumor metabolites like lactate and cytokines disrupt circadian regulation through feedback loops. The deacetylation of BMAL1 by lactate/interleukin-1 beta (IL-1β) amplifies LDHA expression and tumor growth [140]. Hypoxia stabilizes HIF-1α–BMAL1 interactions to suppress mitochondria and enhance glycolysis [42,141]. Systemically, breast cancers remodel liver metabolism via signal transducer and activator of transcription 3 (STAT3)-suppressor of cytokine signaling 3 (SOCS3)-AMPK to create metastatic niches [42,142]. Lung cancers induce CLOCK/BMAL1-mediated hepatic inflammation to evade immunity [42,143]. Such cross-organ circadian reprogramming highlights the integrated metabolic–immune crosstalk in cancer initiation and progression.

## 4. Targeted Intervention Strategies for Spatiotemporal Heterogeneity

Spatiotemporal heterogeneity in tumors arises from metabolic gradients (hypoxic cores to oxygenated rims), stromal interactions, and clonal evolution, creating distinct therapeutic microenvironments. In this section, we evaluate compartment-specific targeting strategies to address the spatial stratification of treatment-resistance mechanisms.

### 4.1. Compartment-Specific Metabolic Targeting

Hypoxic tumor cores exhibit glycolytic dependency mediated by HIF-1α stabilization, targetable through HIF-1α inhibitors [144] (adamantane derivatives [145], MO-2097 [146], NLG207 [147], NB-5-MT [148]), combined with glycolytic enzyme blockade (HK2 inhibitor PF-04691502 [149], LDHA inhibitor FX-11 [150,151]). The oxygen-rich perivascular niche demonstrates GLUT1-mediated glucose addiction, sensitive to GLUT1 inhibitors (BAY-876 [152,153], WZB117 [154]), MCT1 inhibitors (AZD3965 [155], AR-C155858 [156]), and the dual MCT1/MCT4 inhibitor (syrosingopine [157]), further impairing lactate efflux.

Metastatic sites develop organ-specific adaptations: brain metastases utilize ACSS2-dependent lipid metabolism inhibited by AD-5584 [158]; lung metastases are vulnerable to PGC-1α inhibitors (SR18292) [159,160] and PRDX2 blockade (Conoidin A) [161]; liver metastases depend on PDK1-regulated glycolysis targeted by the DCA/CPT combination [162]; and bone metastases engage osteoclast metabolic symbiosis disrupted by the PHGDH inhibitor NCT-503 [96,163], MCT1 inhibitors, or cathepsin K blockade (odanacatib) [97,164].

### 4.2. Temporal Modulation of Metabolic Evolution

Therapeutic strategies must address the dynamic metabolic shifts during tumor progression. Table 4 shows inhibitors targeting glycolysis and the latest drug developments. Early carcinogenesis involves c-Myc-driven glycolytic reprogramming inhibited by c-Myc inhibitors (OmoMYC [165], EN4 [166]). G6PD inhibitors (DHEA [167,168], 6-AN [169]) counteract oxidative stress resistance in p53-deficient cancers by blocking NADPH/GSH production. Emerging compounds (THDOC, G6PDi1 [170]) show improved specificity but require tumor-selective delivery strategies to mitigate normal cell toxicity. Metastatic progression requires EMT–glycolysis pathway disruption through agents like GNE-140 and KIS37 [171,172].

Therapeutic targeting of metabolic reprogramming overcomes drug resistance in cancer, including glycolytic inhibition (2-DG [173], oxamate [174]) and OXPHOS disruption (metformin [175], CB-839 [176]). Circadian-targeted therapies (REV-ERB agonists [177,178], casein kinase 2 (CK2) inhibitors [138,179]) disrupt tumor metabolic rhythms and enhance chemoradiation efficacy in multiple cancers.

**Table 4:** Targeting glycolysis inhibitors and the latest drug developments.

Category	Target	Mechanism of Action	Representative Drugs	Development Status
Glycolytic Key Enzyme Inhibitors				
GLUT Inhibitors	GLUT1, GLUT3	Block glucose uptake; suppress glycolysis initiation.	2-Deoxyglucose (2-DG), WZB117 [154]	Preclinical
Hexokinase (HK) Inhibitors	HK2, HK3	Inhibit glycolysis initiation; reduce ATP/lactate production.	HK2 siRNA [180], 3-Bromopyruvate [181]	Preclinical
Pyruvate Kinase M2 (PKM2) Inhibitors	PKM2	Block nuclear PKM2; inhibit HIF-1α/TGF-β-driven EMT.	Compound 3K [182]	Preclinical
Lactate Dehydrogenase A (LDHA) Inhibitors	LDHA	Suppress lactate production; reverse acidic TME and apoptosis resistance.	FX11 [183], GNE-140 [184]	FX11: Preclinical; GNE-140: Phase I (Breast/Pancreatic Cancer)
Pyruvate Dehydrogenase Kinase (PDK) Inhibitors	PDK1, PDK4	Restore OXPHOS; inhibit glycolysis-dependent EMT.	DCA [185], KIS37 (Cryptotanshinone) [186]	DCA: Phase II (NCT01111097); KIS37: Preclinical
Other Glycolysis-Related Inhibitors				
Vitamin C (High-dose IV)	Indirect (GAPDH)	Depletes NAD+; disrupts microtubule dynamics; inhibits migration.	Ascorbate + Gemcitabine [187]	Phase I/II (NCT02905578, Pancreatic Cancer)
Aldolase A (ALDOA) Inhibitors	ALDOA	Block glycolysis intermediates; suppress cytoskeletal remodeling.	Raltegravir [188]	Preclinical
Enolase 1 (ENO1) Inhibitors	ENO1	Inhibit glycolysis; regulate PI3K/AKT signaling.	ENO1 DNA vaccine [189,190]	Preclinical
Latest Developments				
2-DG + Metformin	Glycolysis/AMPK	Synergistically inhibits EMT and stemness in glioblastoma.	2-DG + Metformin [191]	Preclinical
DCA (Dichloroacetate)	PDK1–EGFR axis	Reverses cisplatin resistance in ovarian cancer.	DCA + Cisplatin [192]	Phase II (NCT01111097)
Repurposed Drugs				
Metformin	AMPK/mTOR	Indirectly inhibits glycolysis and EMT.	Metformin + Chemotherapy [193]	Phase II (NCT01864096, Breast Cancer/Glioblastoma)
Simvastatin	HMG-CoA reductase	Downregulates CXCR4, vimentin; inhibits TGF-β-induced EMT.	Simvastatin [194]	Phase II (NCT00944463, Pancreatic Cancer; NCT02161822, Advanced Rectal Cancer)
Immune-Metabolic Synergistic Approaches				
MCT4 Inhibitors	Lactate export	Block MCT4-mediated lactate efflux to restore T-cell function.	AZD3965 [195,196], Diclofenac [197,198], Syrosingopine [157,195]	Clinical (AZD3965: Phase I/II)
GLUT1 Inhibitors	Glucose uptake	Inhibit GLUT1-mediated glucose transport to counteract TME starvation.	BAY-876 [152], Phloretin [199], Glutor [200]	Preclinical
LDHA Inhibitors	Lactate production	Suppress LDHA activity to reduce immunosuppressive lactate accumulation.	Oxamate [174], Compound 7 [201,202]	Preclinical (Compound 7: Phase I)
GPR81 Antagonists	Lactate signaling	Block GPR81-mediated immunosuppressive signaling in dendritic cells.	3-OBA [203]	Preclinical
SREBP2 Inhibitors	Lipid metabolism	Inhibit SREBP2 to disrupt lactate signaling in antigen-presenting cells.	Fatostatin [63], Botulin [204]	Preclinical
PI3K Inhibitors	Metabolic–immune crosstalk	Dual inhibition of PI3K-AKT-mTOR pathway and lactate signaling.	Copanlisib [205], Duvelisib [205]	Clinical (Copanlisib: FDA-approved for lymphoma)
mTOR Blockers	Immune metabolism	Suppress mTOR-driven metabolic reprogramming in Tregs.	Everolimus [206], CC-223 [207]	Clinical (Everolimus: Approved for multiple cancers)

Note: GLUT, glucose transporter; HK2, hexokinase 2; HK3, hexokinase 3; HIF-1α, hypoxia-inducible factor 1 alpha; TGF-β, transforming growth factor beta; TME, tumor microenvironment; OXPHOS, oxidative phosphorylation; EMT, epithelial-mesenchymal transition; GAPDH, glyceraldehyde-3-phosphate dehydrogenase; NAD+, nicotinamide adenine dinucleotide; AMPK, AMP-activated protein kinase; mTOR, mechanistic target of rapamycin; HMG-CoA, 3-hydroxy-3-methylglutaryl-coenzyme A; CXCR4, C-X-C motif chemokine receptor 4; MCT4, monocarboxylate transporter 4; LDHA, lactate dehydrogenase A; GPR81, G protein-coupled receptor 81; SREBP2, sterol regulatory element-binding protein 2.

### 4.3. Immune–Metabolic Synergistic Approaches

The Warburg effect and lactate secretion create an immunosuppressive tumor microenvironment that is targetable through specific inhibitors: MCT4 inhibitors (AZD3965 [195,196], diclofenac [197,198], syrosingopine [157,195]) block lactate export to restore T-cell function; GLUT1 inhibitors (BAY-876 [152], phloretin [199], Glutor [200]) counteract glucose depletion; LDHA inhibitors (oxamate [174], Compound 7 [201,202]) suppress lactate production; GPR81 antagonism (3-OBA) [203] and sterol regulatory element-binding protein 2 (SREBP2) inhibition (fatostatin [63], botulin [204]) disrupt lactate signaling in dendritic cells; PI3K inhibitors (copanlisib [205], duvelisib [205]) and mTOR blockers (everolimus [206], CC-223 [207]) dually target tumor metabolism and immune suppression. These agents show enhanced efficacy when combined with PD-1/PD-L1 inhibitors, offering a multipronged approach to overcome metabolic immunosuppression.

### 4.4. Innovative Approaches for Targeting Metabolic–Immune Crosstalk

Recent breakthroughs in multimodal imaging, organoid models, nanodelivery systems, and artificial intelligence (AI) have enabled novel strategies for the precision modulation of the metabolic–immune microenvironment. Integrated multimodal imaging (PET/MRI/MALDI-TOF MS, NanoSIMS) elucidates the spatial mapping of metabolic heterogeneity from tissue to subcellular levels [208,209,210,211]. Fluorescent biosensors (FLII12Pglu, SoNar) achieve real-time single-cell metabolic profiling [18,212,213]. Organ-on-a-chip (OOC) systems are microfluidic platforms that recapitulate key physiological and pathological features of human organs, integrating living cells within engineered extracellular matrices and dynamic fluid flow to mimic tissue–tissue interactions and organ-level functions [214]. OOC systems interpret tumor microenvironments with vascular networks and immune interactions, enabling personalized drug testing [215,216]. Patient-derived tumor chips accurately predict clinical treatment responses, improving outcomes for cancers such as glioblastoma and colorectal carcinoma [217,218].

Two core nanosystems have been developed to target tumor microenvironment-specific biomarkers: hypoxia- and enzyme-responsive nanoparticles. Hypoxia-responsive nanocarriers with nitroimidazole moieties and pH-sensitive polymers achieve targeted drug release in acidic tumor microenvironments [219,220]. Enzyme-responsive nanoparticles (GOx-MnO_2_ [221], HAase-AuNR@HA-DC [222], MMP-liposomes [223]) enable precise drug release by responding to hyaluronidase, glucose oxidase, or matrix metalloproteinases in the microenvironment.

Machine learning analyzes tumor metabolism from PET scans and mass spectrometry data to predict treatment response. The REKINDLE model [224] uses reinforcement learning to simulate tumor metabolic adaptation to inhibitors. Machine learning applied to 13C-glucose tracing data predicts optimal drug timing in glioblastoma by disrupting the OXPHOS–glycolysis balance [225]. Supervised machine learning algorithms integrates multi-omics data for personalized regimens [226,227] and temporal metabolic modeling guides therapy adjustments in pancreatic cancer [228]. These technologies collectively address spatial and temporal heterogeneity, enabling targeted interventions that account for tumor evolution and microenvironment interactions.

## 5. Challenges and Future Perspectives

Although significant advances have been made, fundamental questions remain regarding the spatiotemporal regulation of tumor metabolism. Key unresolved issues include: (1) the molecular basis of metabolic heterogeneity across different cancer types; (2) the adaptive mechanisms underlying resistance to metabolism-targeted therapies. A key challenge is developing spatiotemporally precise metabolic modulation, which is a prerequisite for effective targeting.

### 5.1. Current Challenges and Technical Limitations

Current metabolic imaging technologies—from clinical FDG-PET to research-grade hyperpolarized ^13^C-MRI (1–2 mm resolution) and mass spectrometry imaging (50–100 μm)—face inherent trade-offs between spatial resolution and throughput. These limitations constrain the analysis of metabolic heterogeneity at cellular scales. Existing model systems, including patient-derived organoids lacking stromal components, and humanized mice with species-specific metabolic discrepancies, further complicate translational research. The integration of multi-omics data remains challenging, with current methods matching only 50–60% of cells across adjacent tumor sections. Advancing the field requires coordinated development of next-generation imaging, improved computational models, and more physiologically relevant experimental systems.

### 5.2. Clinical Translation Barriers

Metabolic biomarkers have two key limitations: poor specificity of circulating metabolites and unresolved spatial metabolic heterogeneity in tissue biopsies. Exosomal liquid biopsies and PET–genomic integration may improve monitoring. Drug delivery challenges still persist, such as nanocarriers facing stromal barriers. Metabolic-drug combinations risk, genome-scale modeling, and intervention atlases could optimize strategies. Moreover, current metabolic-drug combinations lack rationale, potentially causing antagonism, for example, glycolysis attenuates the efficacy of checkpoint immunotherapy. Computational modeling (genome-scale metabolic models) and systematic atlases of metabolic-drug interactions are needed to identify synergistic sequences and guide clinical translation.

### 5.3. Future Directions

Spatial multi-omics (multiplexed error-robust fluorescence in situ hybridization, Visium, imaging mass spectrometry) reveals new targets at a single-cell resolution. Standardized protocols and lower costs will soon enable metabolic atlases for precision therapies. Targeting microbiome-derived metabolites (e.g., short-chain fatty acids) and intratumoral microbes (Fusobacterium) via probiotics or phage editing may overcome immunosuppression and improve therapy responses. Expanding AI-based digital twins with continuous metabolic monitoring could achieve >75% prediction accuracy, enabling automated therapy optimization across cancer types. The “metabolic clock” framework will combine spatial tumor targeting, circadian timing, and systemic coordination using molecular profiling and adaptive algorithms for precise metabolic therapy.

## 6. Conclusions

Recent advances in single-cell and spatial omics have transformed our understanding of tumor glucose metabolism, revealing dynamic spatiotemporal regulation and metabolic plasticity. These findings establish direct links between glycolytic flux, epigenetic reprogramming, and immune evasion. Clinically, the “metabolic clock” concept provides a framework for timed therapeutic interventions. The remaining challenges include developing tumor-specific inhibitors and understanding metabolic crosstalk in resistant niches. Future work must bridge these mechanistic insights into clinical applications through rigorous translational studies.

## Figures and Tables

**Table 2:** Metabolic adaptations underlying acquired resistance to cancer therapies.

Therapeutic Context	Resistance Mechanism	Key Metabolic Alterations	Associated Biomarkers/Pathways
Gefitinib-Resistant *EGFR*-Mutant NSCLC [35]	Enhanced Glycolysis	Upregulation of GLUT1, HK2, LDHA	Increased lactate production
Vemurafenib-Resistant *BRAF*-Mutant Melanoma [36]	Enhanced Glycolysis	Upregulation of GLUT1/3, HK2 via MEK/ERK signaling	Elevated glucose uptake and glycolytic flux
Trastuzumab-Resistant HER2+ Breast Cancer [37]	Enhanced Glycolysis	PFKFB3-mediated fructose-2,6-bisphosphate synthesis	Increased glycolytic activity
Osimertinib-Resistant NSCLC [38]	Mitochondrial Reprogramming	Enhanced OXPHOS	Increased OXPHOS activity
BRAF Inhibitor-Resistant Melanoma [39]	Mitochondrial Biogenesis	PGC-1α-mediated increase in mitochondrial density and oxidative capacity	Elevated cristae density, increased OXPHOS
Immunotherapy Resistance (e.g., PD-1 Inhibition) [40]	Metabolic Reprogramming Linked to Immune Evasion	Selection for clones with enhanced OXPHOS, co-expression of TIM-3	Link between metabolic reprogramming and immune evasion

Note: EGFR, epidermal growth factor receptor; NSCLC, non-small cell lung cancer; GLUT1, glucose transporter 1; HK2, hexokinase 2; LDHA, lactate dehydrogenase A; BRAF, B-Raf proto-oncogene, serine/threonine kinase; HER2, human epidermal growth factor receptor 2; PFKFB3, 6-phosphofructo-2-kinase/fructose-2,6-biphosphatase 3; OXPHOS, oxidative phosphorylation; PGC-1α, peroxisome proliferator-activated receptor gamma coactivator 1-alpha; PD-1, programmed cell death protein 1; TIM-3, T-cell immunoglobulin and mucin-domain containing-3.

**Table 3:** Hypoxia-induced metabolites function as epigenetic modulators.

Metabolite	Target Enzymes	Epigenetic Effects	Key Mechanisms
Lactate [51,52]	HDACs, GPR81	Increased H3K18 lactylation	Lactate promotes the epithelial–mesenchymal transition of liver cancer cells via TWIST1 lactylation.
Succinate [53]	PHDs, KDMs	DNA hypermethylation	Succinic acid stabilizes HIF-1α activity by inhibiting its degradation. Succinate promotes angiogenesis by inducing the expression of VEGF.
Fumarate [56]	TETs, PHDs	Histone succinylation	The accumulation of fumaric acid results in pseudohypoxia by stabilizing the transcription factor HIF-1α and DNA hypermethylation, and inhibiting enzymes of the 2OGDD family.
2-HG [57,58]	FTO, ATP synthase	Decreased m^6^A modification	Selective inhibition of the m6A epitranscriptomic regulator FTO attenuates growth in IDH1wt glioma.

Note: HDACs, histone deacetylases; GPR81, G-protein-coupled receptor 81; TWIST1, twist family bHLH transcription factor 1; PHD, prolyl hydroxylase; KDM, histone lysine demethylase; HIF-1α, hypoxia-inducible factor 1 alpha; VEGF, vascular endothelial growth factor; TET, ten–eleven translocation methylcytosine dioxygenases; 2OGDD, 2-oxoglutarate-dependent dioxygenases; 2-HG, 2-hydroxyglutarate; FTO, fat mass and obesity-associated protein; ATP, adenosine triphosphate; IDH1wt, isocitrate dehydrogenase 1 wild type.
